# The dual system model of distraction: explaining the cognitive mechanism of distraction

**DOI:** 10.3389/fpsyg.2025.1632165

**Published:** 2025-10-16

**Authors:** Chenghao Wang

**Affiliations:** School of Education, Tangshan Normal University, Tangshan, Hebei, China

**Keywords:** distraction, attention, model, learning, attention control, attention capacity, cognition

## Abstract

In this conceptual analysis, the theoretical foundations of distraction are examined, with particular emphasis on its definitional ambiguity and the absence of systematic models. The dual system model of distraction is introduced, delineating two complementary mechanisms: the distraction capacity system, a limited unconscious mechanism that automatically resists task-irrelevant input, and the attention control system, a conscious mechanism that reorients focus when distraction exceeds capacity. The first aim is to situate distraction in relation to established attention theories, showing how filter, attenuation, and capacity models frame distraction only indirectly. A review of contemporary distraction-related accounts and findings, such as goal interference and resource availability models, is then provided to demonstrate the lack of a unified framework. On this basis, the dual system model is explained as accounting for inattentional blindness, inattentional deafness, the state of flow, and the transition from recognizing distraction to re-engaging with the primary task. The model’s relevance for educational contexts is outlined, where technology-induced distractions present a pressing challenge for sustained attention. Finally, it is argued that the dual system model serves as an epistemic framework that integrates unconscious resistance and conscious control, thereby providing a conceptual foundation for future empirical research and applied interventions in distraction-prone environments.

## Introduction

The distractions that students encounter in daily learning contexts pose persistent challenges for both parents and instructors, who often observe how focused attention can shift from learning tasks to competing, task-irrelevant stimuli. Notifications from smartphones, background music, conversations among others, and even task-irrelevant thoughts are pervasive sources of distraction that significantly impair learning performance, underscoring the difficulty of sustaining focused attention in environments saturated with potential distractions (de la Mora Velasco et al., 2023; Forster, 2013; Rozgonjuk et al., 2019). With the increasing severity of distraction, understanding the cognitive mechanism of distraction is both critical and foundational. However, unlike the decades of prosperous studies on attention, distraction, an equivalently important cognitive construction, has long been omitted and taken for granted. Until today, no widely recognized theory or model that specifically explains the cognitive process of distraction, which inevitably hinders our understanding of distraction and affects conducting distraction-related studies. Therefore, despite its widespread use in research, the concept of distraction remains theoretically underdeveloped, resulting in inconsistent and restricted applications (Schmidt, 2020). For example, distraction, more specifically digital distraction, is defined by Flanigan and Kim (2022) as “the misuse of mobile technology for leisure purposes while attending to academic tasks inside or outside of the classroom,” which solely focuses on the distractive influence from mobile technology. By contrast, *driver distraction* has been described as “occurring when a driver’s attention is, voluntarily or involuntarily, diverted away from the driving task by an event or object to the extent that the driver is no longer able to perform the driving task adequately or safely” (Young et al., 2007), emphasizing the attentional shift that compromises driving performance. Although these operationalizations capture domain-specific manifestations, both are ultimately grounded in the theoretical framework of attention rather than distraction itself. This further underscores the need to establish distraction as a distinct construct by investigating its underlying cognitive mechanism.

In recent years, scholarly interest in distraction has grown markedly, driven in large part by the escalating prevalence of technology-induced distractions from smartphones, tablets, and laptops (Brady et al., 2022; Göl et al., 2023; Zhang et al., 2022). A substantial body of empirical evidence indicates that the use of digital devices during class or online learning undermines students’ academic performance and their capacity to sustain focused attention (Blasiman et al., 2018; Fook et al., 2021; Martin et al., 2025; Zhang et al., 2022). Reflecting this trend, an increasing number of studies employ the term *digital distraction* to describe task-irrelevant device use (Flanigan and Kim, 2022) or media multitasking behaviors (Aagaard, 2019). Beyond educational contexts, neurological research further demonstrates the detrimental effects of distraction on task performance, underscoring its significance within cognitive neuroscience (Wei et al., 2024). Similarly, the issue of distracted driving has been extensively examined from both neurophysiological and applied perspectives, with recent advances such as the EEG-based “Distraction Index” developed by Ronca et al. (2024), which quantifies driver distraction under varying road and traffic conditions by analyzing EEG frequency bands. EEG was also utilized by Kuang et al. (2025) for developing a measurement that assesses distraction among coal mine workers. Collectively, these lines of inquiry highlight distraction as an increasingly salient research topic across multiple domains. Nevertheless, the continued absence of distraction-specific theories and models poses a substantial obstacle to understanding its underlying cognitive processes and generalizing empirical findings. Addressing this gap requires the development of a theoretical framework capable of systematically explicating the mechanisms of distraction, thereby establishing a foundation for advancing both conceptual and empirical research.

To counter and minimize the detrimental effects of distraction, various practical approaches have been developed. One widely recognized strategy is the incorporation of rest breaks, which is an effective approach to restore depleted attentional resources and re-establish focused attention on tasks, as supported by Attention Restoration Theory (Schumann et al., 2022). Meanwhile, with the growing prevalence of online learning and self-directed study, the design of learning environments that minimize the influence of distraction has become another important direction (Lin et al., 2022). Nevertheless, the absence of a comprehensive understanding of the underlying cognitive mechanisms of distraction continues to limit the development of more systematic and theoretically grounded interventions.

For advancing the psychology theory in distraction and moving the field forward, this article introduces the dual system model of distraction as a framework to comprehensively elucidate the underlying cognitive mechanism of distraction. This article begins by outlining the current contentions surrounding the concept of distraction and introduces three central questions for the development of a distraction theory or model: (1) How can the underlying mechanisms of inattentional blindness and inattentional deafness be explained? (2) How is the experience of flow accounted for by the same mechanisms? (3) What is the cognitive processes underlining the transition from consciously recognizing a distraction to reorienting attention toward the primary task? Answering these three questions leads to the reveal of the cognitive process of distraction and the development of a novel distraction model. Subsequently, the proposed dual system model has been introduced as a unified model of distraction-aiming at addressing the three central questions, explicating its underlying mechanisms, and articulating its theoretical alignment with attention.

The primary objective of the proposed model is to comprehensively explain the cognitive mechanism of distraction under the perspective of distraction. Furthermore, the proposed model aims to introduce the theoretical significance of distraction within the broader domain of attention by elucidating its theoretical relevance to attention and its critical distinctions from attention. Finally, offering a comprehensive account of the functional orientation of distraction, emphasizing its contributions in educational settings.

## The contested definition of distraction

Despite the growing significance of attention in both cognitive and educational psychology, distraction received significantly less scholarly focus, which hindered the development of an academic field dedicated to the study of distraction. Although distraction is widely referenced across psychological literature, it remains theoretically underdeveloped, lacking a coherent conceptual framework and comprehensive theoretical grounding. As a result, persistent inconsistencies continue to surround its definition and interpretation.

Scholars from different fields have proposed various definitions of distraction. In the field of driving behavior, Streff (2000) defined driver distraction as “a shift in attention away from stimuli critical to safe driving toward stimuli that are not related to safe driving.” In the field of education, Flanigan and Kim (2022) defined digital distraction as “the misuse of mobile technology for leisure purposes while attending to academic tasks inside or outside of the classroom.” In the workplace, distraction was defined by Sanders and Baron (1975) as “an employee’s inability to perform their assigned tasks.” These definitions of distraction are specific to their respective fields and lack generalizability. Additionally, Smiley (2005) proposed a simple definition of distraction as “misallocated attention,” while Gazzaley and Rosen (2016) concisely defined distraction as “task-irrelevant information.” Although these two broader definitions offer a more general perspective, they do not adequately explain the mechanism underlying distraction.

Wang (2022) proposed a comprehensive definition of distraction after systematically reviewing and synthesizing definitions across various disciplines. Distraction was defined as “diverting attention from a primary task to a secondary task, or allocating partial attention to task-irrelevant information, resulting in negative effects on task performance.” This definition clarifies the manifestation of distraction and emphasizes its detrimental impact on task execution. However, it remains primarily descriptive and does not address the cognitive mechanisms underlying distraction. To reconcile definitional inconsistencies and deepen our understanding of distraction, it is essential to investigate its cognitive processes and develop a theoretical model that explains how distraction operates.

In addition to the definitional inconsistencies surrounding distraction, scholars have also held divergent views regarding what distraction represents, often using related concepts interchangeably. For instance, Blasiman et al. (2018) and Mokhtari et al. (2015) conceptualized multitasking as a form of distraction, treating the two constructs synonymously in their research. In contrast, Unsworth and McMillan (2017) and Risko et al. (2013) equated distraction with mind-wandering, emphasizing its relation to attentional control and executive functioning. Meanwhile, Flanigan et al. (2022) identified digital device usage—particularly smartphones and laptops for non-academic purposes—as a predominant source of classroom distraction. Rusz et al. (2020) associated distraction with reward mechanisms, suggesting that reward sensitivity contributes to attentional shifts. Similarly, Brady et al. (2021) introduced the concept of “physiology” to account for distraction arising from students’ physical states, noting that adverse physiological conditions can impair attentional focus. These varied conceptualizations underscore the need for a unifying theoretical framework or model of distraction—one that can systematically elucidate its cognitive underpinnings and classify its diverse manifestations.

The ongoing debates surrounding both the definition of distraction and its conceptual representation underscore the persistent ambiguity of the construct. This lack of clarity highlights the urgent need for a coherent theory or model of distraction to guide future research and inform practical applications.

## Conceptualizing distraction through attention models and distraction-related models

Building upon existing theoretical perspectives, the presence of a complex cognitive process underlying distraction can be reasonably inferred, providing a conceptual foundation for the proposed model. Two categories of theoretical models need to be comprehensively reviewed, attention models and distraction-related models, offering valuable insights into understanding the cognitive process of distraction.

### Attention models and distraction

Three widely recognized attention models—Broadbent’s Filter Model, Treisman’s Attenuation Model, and Kahneman’s Capacity Model—have been reviewed to conceptualize distraction from the perspective of attention. Rather than detailing the entire theoretical analysis, the main conclusions are summarized concisely. Broadbent’s Filter Model conceptualizes distraction as the intrusion of task-irrelevant stimuli that inadvertently passes through the cognitive filter, thereby diverting attention away from the primary task toward unrelated inputs (Broadbent, 1958). This model offers one of the earliest theoretical accounts of the cognitive mechanism underlying distraction.

Treisman’s Attenuation Model extends this understanding by emphasizing the involuntary perception and partial processing of unattended stimuli through an attenuation mechanism (Treisman, 1964). According to this model, task-irrelevant stimuli are not entirely filtered out but instead weakened; those with sufficient salience or low recognition thresholds can surpass the attenuation threshold and enter the attended channel, thereby capturing attention. While this conceptualization aligns with Broadbent’s to some extent, Treisman’s model further clarifies how certain distractors are processed and become consciously accessible.

Kahneman’s Capacity Model provides a foundational and resource-based framework for understanding attention, proposing that attentional resources are limited and must be allocated among competing demands (Kahneman, 1973). From this perspective, distraction occurs when insufficient attentional resources are allocated to the primary task and excessive resources are unintentionally directed toward irrelevant stimuli, resulting in a shift of attention. Kahneman’s model offers a more comprehensive explanation by linking distraction to the dynamics of attentional allocation.

Together, these three models significantly contribute to the theoretical understanding of distraction and continue to exert substantial influence on both empirical and conceptual research in the field. However, contemporary distraction-related studies remain largely grounded in the attention perspective—interpreting distraction primarily through attention theories and models. While attention and distraction are complementary constructs, limiting distraction research to the attention framework may obscure unique features of distraction. A shift toward a dedicated distraction perspective is necessary to more fully elucidate the cognitive mechanisms and theoretical conceptualization of distraction itself.

### Distraction-related models and distraction

Reviewing distraction-related models is equivalently important to reviewing attention models. However, given that no theory or model of distraction has achieved widespread recognition, the representative Gazzaley and Rosen’s goal interference model and Taatgen’s resource-availability model of distraction have been examined for further conceptualizing distraction.

Gazzaley and Rosen’s goal interference model indicates goal interference occurs when experience things that hinder or impede goal completion process, which is a concept closely aligned with distraction (Gazzaley and Rosen, 2016). Gazzaley and Rosen’s model distinguishes two primary types of interference—internal and external—each divided into distractions (involuntary) and interruptions (voluntary). Internal distraction involves unintentional mind-wandering, often triggered by boredom, whereas internal interruption entails deliberate multitasking with internal tasks, such as checking a smartphone during class. External distraction arises from involuntary shifts of attention to irrelevant sensory stimuli, such as a ringing phone, while external interruption occurs when individuals voluntarily redirect attention to an external task, such as eavesdropping on a conversation.

This taxonomy offers a comprehensive classification of interference types, advancing the theoretical understanding of distraction.

The theoretical foundation of Taatgen’s resource-availability model of distraction is largely based on the limited attention capacity of Kahneman’s model, positing the occurrence of distraction is because of the insufficient attentional resources occupied by the primary task (Taatgen et al., 2021). The model conceptualizes distraction by examining the cognitive mechanisms underlying one of its most significant forms—multitasking—arguing that multiple tasks can be processed in parallel when sufficient resources are available for each and task demands do not overlap. Although derived from an attention-based framework, Taatgen’s model advances the understanding of distraction by elucidating the allocation of attentional resources from the perspective of distraction rather than attention.

## Contemporary methods for measuring distraction

In addition to resolving the contested definition of distraction, summarizing how distraction is observed and further how can be measured is also important for advancing the understanding of distraction. Without systematic measurements, the concept of distraction remains vague, interpreted inconsistently across disciplines. Therefore, discussing current methods that measures distraction is beneficial for obtaining a comprehensive understanding of distraction.

One important approach to examining distraction is through objective measures of attentional performance. Distraction can be observed in lapses of task focus, quantifiable costs in response speed, accuracy, and variability, as well as in the subjective experience of attentional diversion. Weissman et al. (2006), using functional magnetic resonance imaging (fMRI) during the Sustained Attention to Response Task (SART), demonstrated that momentary lapses of attention were associated with reduced activation in prefrontal and anterior cingulate regions. Their findings suggest that the experience and detrimental effects of distraction can be measured through cognitive tasks that directly measure sustained attention. This study evidence aligns with the principles of Attention Restoration Theory, which posits that the maintenance of focused attention becomes increasingly difficult over time, and that rest breaks are essential for replenishing attentional resources and sustaining performance (Schumann et al., 2022). Complementary evidence comes from Andrillon et al. (2021), who monitored participants’ performance on the SART using EEG. They observed that declines in task performance were accompanied by slowing neural activity and the emergence of mind wandering. Together, these findings underscore that distraction can be effectively operationalized and measured through attention-related cognitive tasks.

Another important approach to examining distraction is through subjective measurement. Self-report has been widely employed in attention and distraction research because it provides direct access to individuals’ subjective experience of attentional states. While objective measures of attentional performance yield quantifiable and comparable results, subjective reports remain valuable as they capture the phenomenological aspects of distraction that cannot be fully represented in behavioral or neural indices. Distraction can be measrured through individuals’ introspective accounts of attentional lapses, perceived difficulties in maintaining focus, and the subjective experience of mind wandering. For example, Smallwood and Schooler (2006) employed probe-caught techniques during SART, which demonstrated that participants’ self-reports of being off-task were strongly associated with concurrent declines in task performance. Their findings suggest that subjective reports provide valid indicators of attentional diversion and offer a complementary perspective to performance-based measures. Supporting this perspective, Kane et al. (2007) used experience-sampling methodology in daily life and found that self-reported mind-wandering experience contributes to measure attention control and working memory performance. Together, these findings indicate that measuring distraction through subjective reports is not only methodologically feasible but also theoretically valuable.

Although distraction is estimated based on measures of attention, contemporary methods for measuring distraction remains limited. Current methods emphasize lapses of task focus or task performance, but they fail to consider the underlying cognitive mechanism of distraction. In particular, the role of unconscious processes that automatically filter or inhibit irrelevant stimuli, as well as the conscious control mechanisms that reorient attention once distraction occurs, are rarely incorporated into existing measurement frameworks. Without integrating both the implicit capacity to resist distraction and the explicit strategies of attentional control, attempts to quantify distraction risk providing only a partial account of the phenomenon. To advance the understanding of distraction, research must move beyond surface-level objective and subjective indices toward a more mechanistic framework that develops methods capable of elucidating how unconscious resistance and conscious control jointly shape its dynamics.

## The cognitive process of distraction: three questions raised

Both attention models and distraction-related models have substantially contributed to the conceptualization of distraction, with the latter offering the valuable shift of examining distraction from its own perspective rather than solely through the lens of attention. However, in the absence of a unified theoretical framework for distraction, its cognitive mechanisms continue to be predominantly explained using attention models. To develop a distraction model that accounts for these mechanisms from the perspective of distraction, three key questions must be addressed: (1) How can the underlying mechanisms of inattentional blindness and inattentional deafness be explained? (2) How might the experience of flow be accounted for by the same mechanisms? (3) What cognitive processes underline the transition from consciously recognizing a distraction to reorienting attention toward the primary task?

The first question pertains to the phenomena of inattentional blindness and inattentional deafness. These occurrences are characterized by an almost complete lack of influence from distractions. This raises the question: how can the cognitive processes underlying these phenomena be conceptualized within the framework of distraction? Specifically, why, in certain circumstances, are we able to maintain focused attention on a primary task without being impaired by perceivable distractions?

This inquiry further leads to a related question concerning the state of flow. The state of flow represents the full immersion to a primary task and the total resistance to distractions, which significantly enhances performance and efficiency on the primary task (Harris et al., 2023; Jinmin and Qi, 2023). Achieving the state of flow, therefore, implies an increased capacity to resist perceived distractions. In other words, when in the state of flow, distractions that would ordinarily divert attention from the primary task no longer have the same impact. For example, individuals may miss notification sounds from their cellphone when completely immersed in completing an assignment. In contrast, when attention is not fully devoted to the task, these same notifications can easily disrupt focus and prompt the individual to check their phone. This contrast in the ability to resist distractions highlights the importance of exploring the underlying cognitive processes and mechanisms of distraction.

Although individuals can unconsciously resist perceived distractions, not all distractions can be successfully resisted. Pain serves as a prominent example of a perceived distraction that demands immediate attention and interrupts ongoing tasks (Öhman, 1979). For example, a student might choose to persist in completing a final exam despite experiencing a severe stomachache. According to conventional understandings of distraction, it would be expected that the student’s attention would be consistently disrupted by the ongoing stomachache, making it difficult to maintain focused attention on the exam. However, in practice, individuals are often able to tolerate the discomfort and refocus on the task, suggesting the existence of a cognitive mechanism that allows for the resolution of such distractions. This raises an important question: what is the cognitive process that underlies the transition from consciously recognizing the distraction to refocusing on the primary task?

## Investigating the cognitive process of distraction in educational settings

While investigating the cognitive process of distraction through three central questions, it is essential to account for its variability across different contextual settings. Attempting to propose a single, universal model that explains the cognitive mechanisms of distraction in all contexts may be overly idealistic and theoretically limiting. In the absence of empirical evidence supporting such generalizability, the validity of a context-independent explanation remains uncertain and requires further investigation. Conversely, investigating the cognitive process of distraction within specific contextual settings offers more nuanced and practically meaningful insights, thereby strengthening the conceptual foundation and empirical support for the proposed model. Compared to driving and workplace environments, educational settings—such as classrooms, libraries, and dormitories—offer a particularly suitable and impactful context for investigating the cognitive processes underlying distraction. This is evidenced by a growing body of research indicating that students are increasingly distracted by digital devices during lectures and while completing academic tasks, highlighting the escalating severity of technology-induced distraction in learning environments (Aaron and Lipton, 2018; Le Roux and Parry, 2022). The suitability of educational contexts for such investigation is further reinforced by the relatively extensive empirical literature in this domain and the societal emphasis on optimizing students’ learning experiences (Flanigan et al., 2022; Le Roux and Parry, 2022; Taneja et al., 2015). Collectively, educational settings are a representative and theoretically informative context for investigating the cognitive process and implications of distraction.

## The dual system model of distraction

The dual-system model aims to comprehensively explain the whole cognitive mechanism of distraction, from the perception of a distraction to being distracted, under the perspective of distraction. Conceptually, rather than positioning this model as entirely distinct from attention, it is more appropriate to regard it as an extension of the current theoretical understanding and consensus on attention. Attention has been widely perceived as a limited capacity that can actively allocate attentional resources through active control, which leans more to the conscious side while the unconscious side has rarely been discussed, especially under the perspective of distraction (Eysenck et al., 2007; Kahneman, 1973; Stiegler, 2010). For example, when students write an essay on cognitive psychology, research often focuses on why they can maintain attention on the writing task, rather than on why they are not distracted by various types of competing stimuli. Examining the cognitive processes underlying distraction extends our understanding of attention from the conscious to the unconscious level. In this sense, the proposed model expands the concept of attention by positioning distraction as a complementary psychological construct, thereby offering a more comprehensive understanding of distraction.

The proposed model is most appropriately characterized as an *epistemic-symbolic model*, meaning that it serves primarily as a conceptual framework aimed at explaining and organizing knowledge about the phenomenon of distraction rather than providing a directly testable or computational account. By adopting this form, the proposed model contributes to the theoretical expansion of attention research, positioning distraction as a complementary construct that interacts with attention at both conscious and unconscious levels.

This model introduces two cognitive systems, the distraction capacity and attention control system, for explaining the unconscious and conscious resistance of distractions, respectively. The distraction capacity represents a limited capacity that automatically and unconsciously resists the negative influence of distractions while focusing on a task, which explains why we can maintain focused attention on a task while surrounded with numerous distractions. In another perspective, distraction capacity also explains the occurrence of both inattentional blindness and inattentional deafness by considering task-irrelevant visual and verbal information is automatically and unconsciously resisted, so people experience no distractive influence from varies distractions while focusing on a task. The attention control system is different from the current understanding of attention control. Instead, it specifically represents the cognitive process of shifting attention from distraction back to primary task after consciously aware of the perceived distraction. As Smallwood and Schooler (2006) indicated that we have a restless mind that keeps wandering, it is inevitable that our attention shifts among tasks and process task-irrelevant information. So, after consciously aware of the distraction, actively shifting our attention from distraction back to primary task is another indispensable cognitive process that significantly contributes to task performance and efficiency.

**Figure 1:**
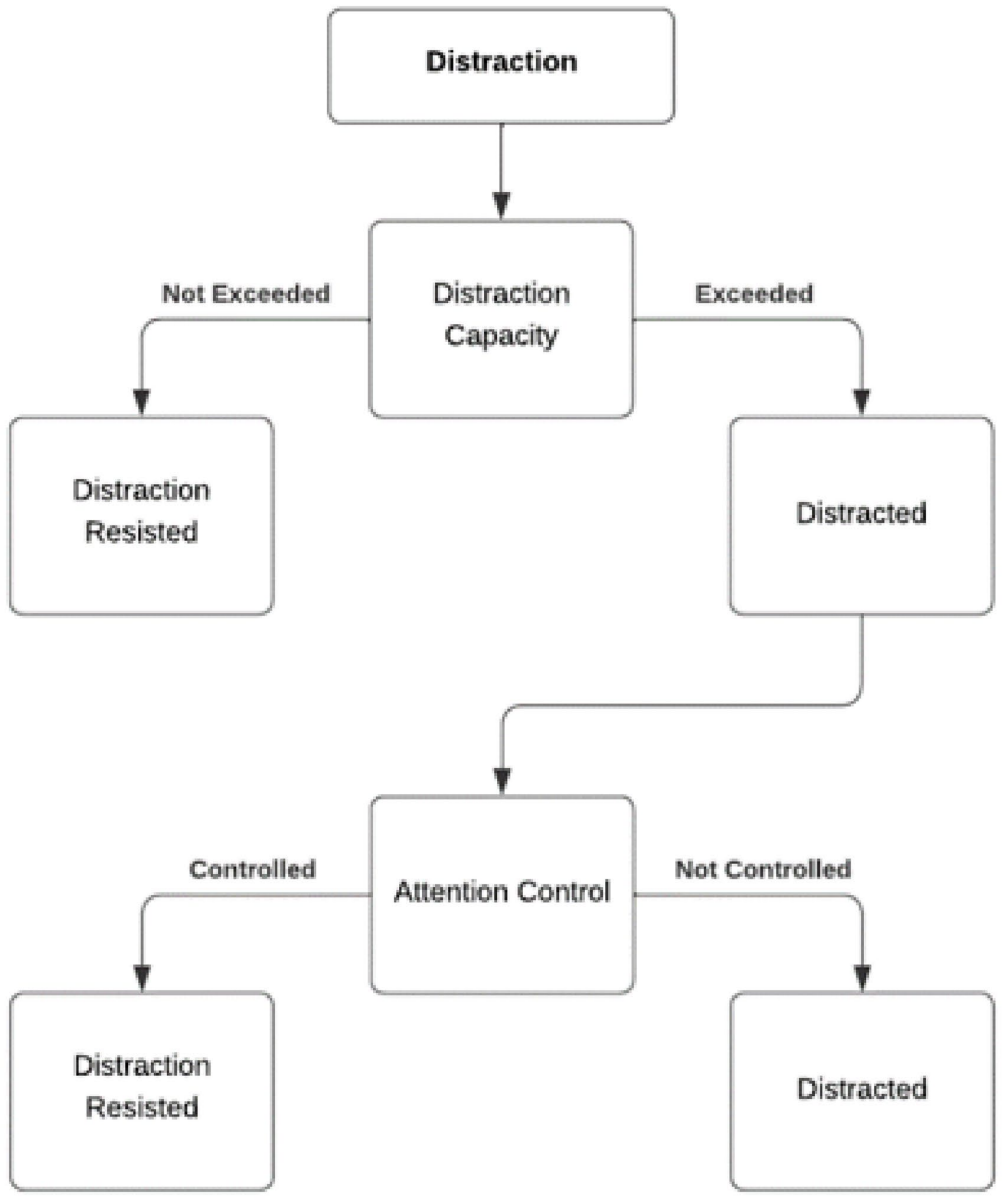
The cognitive process of distraction.

The comprehensive cognitive process of distraction is presented in Figure 1. At the beginning, we have a primary task that needs to be focused on while we perceive all kinds of task-irrelevant distractions in the surrounding environment, including different types of sensory stimuli and task-unrelated thoughts generated within our minds (Kandel et al., 2021; Wong et al., 2022). As we focused on our primary task, certain distractions are resisted within the distraction capacity and have no distractive influence on task performance. Such cognitive system explains how focused attention can be achieved and how the distractive influence of certain distractions is resisted. However, not all distractions can be continuously resisted by distraction capacity. The distractive influence of certain distractions may exceed the distraction capacity, inevitably leading to the conscious awareness of being distracted. Then the attention control system is involved for actively determining whether to ignore this distraction and re-focusing on primary task or relinquishing our focused attention on task and indulging our attention to focus on the perceived distraction. Deciding to re-focus on primary task represents the success of controlling attention and resisting distraction, whereas shifting attention to distraction is a representation of failing to control attention and resist distraction.

Meanwhile, it is extremely important to note that the premise of proposed cognitive process of distraction, which is actively focusing on the primary task. In other words, distraction is only a problem when we need to achieve focused attention on a task. Otherwise, as Eyal (2019) mentioned, you cannot be distracted if you are entertaining. Without the need to focus on a task, the problem of distraction ceases to exist. Therefore, having such a premise is indispensable for understanding the cognitive process of distraction.

## The distraction capacity system

In our daily lives, we constantly hear sounds, see things, and have irrelevant thoughts while working on a task, making completely focus on a task impossible in theory (Schmidt, 2020; Smallwood and Schooler, 2006). But in fact, we are fully capable of focusing on a task without processing these task-irrelevant distractions. For example, looking at a tree through the window while thinking about the possible ideas for your next project. The primary task is thinking about ideas for a project and your attention is maintained on your primary task even if you are looking at the distraction, the tree. So, why can we keep thinking about ideas without being distracted by the tree? And what is the within mechanism of preventing the distractive influence of the tree? I argue that the reason why we can focus on the task and resist distractions is because we have a distraction capacity that automatically resists a certain amount of perceived distractions, thereby the resisted distractions are not being consciously perceived nor even processed, which facilitates to achieve the state of focused attention and minimize the influence of distractions. The mechanism of distraction capacity also supports that the automated tasks should not be considered as a task of multitasking behavior since the distractive influence of the automated tasks has been automatically resisted by the distraction capacity. For instance, while thinking of possible topics of an essay, grabbing coffee cup and drinking coffee. In this example, most times grabbing and drinking coffee is an automated behavior that has no distractive influence on the primary task, thinking of possible topics for the essay. Therefore, the automated task is another piece of evidence that demonstrates the mechanism of distraction capacity.

Distraction capacity also illustrates that the occurrence of distraction accompanies with the conscious awareness of processing task-irrelevant information. In other words, we are only distracted when we are consciously aware that we are processing task-irrelevant information, whereas when the distractions are visually or verbally presented while triggering no task-irrelevant thoughts, we are not considered as being distracted. Such distinction is supported by rigorous theoretical inference. Some widely recognized psychological effects and constructs, including irrelevant sound effect, inattentional blindness, inattentional deafness, and the state of flow, are discussed in the following sections for the comprehensive establishment of distraction capacity.

### The problem of consistent interference and irrelevant sound effect

Completing tasks in a distraction-free environment is an ideal but rarely attainable scenario. Various distractions, commonly referring to consistent interference (Wang, 2022), includes indoor noise (e.g., conversations, kitchen sounds, TV, air conditioning), outdoor noise (e.g., lawnmowers, traffic, wind, construction), and irrelevant objects in the workspace (e.g., car models, pictures, magazines), impairing focused attention and thereby negatively impacting task performance. Studies found the distractive influence of music sounds on the performance of cognitive tasks and studying (de la Mora Velasco et al., 2023; Doyle and Furnham, 2012; Goltz and Sadakata, 2021). Meanwhile, the distractive influence of the presence of distractive objects on task performance has also been supported. Przybylski and Weinstein (2013) found the distractive influence on the quality and connection of participants’ face-to-face conversation under the presence of smartphone, suggesting the importance of minimizing the presence of task-unrelated objects, especially digital devices.

Two widely recognized psychological effects, irrelevant-speech effect and irrelevant sound effect (ISE), are precisely describing such problem of distraction and providing important insights about distraction. Salame and Baddeley (1982) first explored the influence of unattended speech on the short-term memory by presenting nonsense words or meaningful words while memorizing the digits (nine random numbers) and the result found that the participants’ performance of memorizing digits was impaired by the irrelevant sounds, both nonsense words and meaningful words. Another study conducted by Elliott (2002) specifically found the detrimental effect of irrelevant speech was more severe on the performance of children compared to the performance of adults, which directly supported the distractive effect of irrelevant speech on task performance. The irrelevant sound effect is relatively a more generalized effect that discusses the same problem, which refers to the substantial impairment of short-term memory performance when exposed to task-irrelevant sound (Macken et al., 2009). Klatte et al. (2013) concluded that irrelevant sounds, such as chronic noise, have negative effects on children’s cognitive performance. Therefore, the distractive effect of sound distractions on task performance is widely recognized. Wang (2022) further summarized this kind of distraction as “consistent interference,” representing the consistent experience of task-irrelevant distractions, including both verbal distractions and visual distractions. However, another serious problem needs to be asked, how do we maintain focused attention on a task while experiencing consistent interferences and what is the mechanism within?

Based on the consensus that ideal learning environment is rarely attainable and consistent interferences impair focused attention during tasks, it is critical to explore the cognitive process of distraction. But first, the current understanding of the mechanism of ISE needs to be discussed. Currently, the mechanism of ISE has been explained under the perspective of attention and working memory. Meinhardt-Injac et al. (2022) summarized that one reason ISE happens is because irrelevant sounds capture attention and redirect attention from current tasks to verbal distraction which disrupts cognitive performance. According to the theoretical review conducted by Wang (in review), ISE can also be explained by Treisman’s attenuation model of attention as the irrelevant sounds are being perceived and partially processed even attention is directed toward the primary task, suggesting that distractions are constantly being perceived but not all distractions are being processed in parallel with the primary task. Another reason is based on two mechanisms of the duplex-mechanism account, interference-by-process mechanism and attention capture mechanism, indicating ISE occurs when salient stimuli is captured by attention while non-salient stimuli is ignored (Hughes et al., 2013). The widely recognized Baddeley’s multicomponent model of working memory explains ISE based on the mechanism of phonological loop component. Salame and Baddeley (1982) considered irrelevant sounds interfere with the temporal storage of verbal information and the process of articulation, which leads to poorer performance on short-memory tasks. These explanations support that irrelevant sound interferes with the cognitive processing of the primary task and result the negative consequences. However, how consistent interference, such as irrelevant sound, is resisted while focusing on a task has not been discussed, especially from the perspective of distraction.

### Distraction capacity and inattentional blindness

The classic inattentional blindness experiment conducted by Simons and Chabris (1999) perfectly demonstrated how people ignored the person in a gorilla suit who walked across the scene while paying full attention to counting the number of basketballs passes between players. They explained that the occurrence of inattentional blindness is the result of failing to be aware of visual information when focused on a task. But the question of what the cognitive mechanism behind inattentional blindness still needs to be answered. The widely recognized load theory proposed by Lavie et al. (2004) explained the occurrence of inattentional blindness based on the perspective of attention. The task with high cognitive load demands high attentional resources, which may leave insufficient resources for processing irrelevant information. Related to Simons and Chabris’s experiment, counting the number basketball passes is the task with high cognitive load since participants need to consistently maintain focused attention on the basketball. The walking gorilla is irrelevant visual information that has not been processed with attention resource. However, there is no explanation based on the perspective of distraction.

Since the proposed dual system model of distraction is a model that comprehensively explains the cognitive process of distraction, explaining the problem of inattentional blindness based on the perspective of distraction is important. Taking Simons and Chabris’s experiment as an example again. Counting the number basketball passes is the primary task that participants need to focus on, and the walking gorilla is the irrelevant visual information that has been automatically resisted since such distractive influence is within participants’ distraction capacity. Therefore, we experience inattentional blindness because irrelevant information is automatically resisted for favoring the maintenance of focused attention on the primary task.

Besides Taking Simons and Chabris’s experiment, several replication studies found and supported the occurrence of inattentional blindness in different contexts. Hyman et al. (2010) interviewed individuals who walk through the Red Square in Western Washington University and asked whether they noticed a unicycled clown. Compared to individuals without electronics and individuals who listened to music, the individuals who used cell phone were least likely to see the unicycling clown, which proved the existence of inattentional blindness. Furthermore, Drew et al. (2013) studied inattentional blindness through asking radiologists to detect the small abnormalities in lung nodules through carefully searching five lung CTs while a gorilla image was inserted in one of five CTs. The result indicated 83% of radiologists did not see the gorilla and 50% of radiologists failed to report even though they were directly looked at the gorilla’s location though eye-tracking, which further supported the existence of inattentional blindness. Although inattentional blindness has become a widely recognized psychological phenomenon based on current evidence, the cognitive process behind has been rarely discussed. The distraction capacity provides an alternative explanation of inattentional blindness for deepening the understanding of relevant concepts.

### Distraction capacity and inattentional deafness

Similar to the findings on inattentional blindness, the studies on inattentional deafness also found the existence of inattentional deafness in varies contexts. Koreimann et al. (2014) conducted an experiment for investigating whether non-musicians noticed the irrelevant sound (e-guitar solo during the last 20 s) while they were asked to count the number of tympani beats from start to end. The result indicated only one non-musician from the experimental group noticed the e-guitar sole and over half of non-musicians from the control group actively mentioned the e-guitar solo, which suggested focusing on a task accompanies with resisting irrelevant information among non-musicians. Similar, but less extreme result was found among amateur musicians, suggesting counting beats requires less attention resource for musicians and their state of focused attention is hardly maintained for the whole process. Another experiment conducted by Dalton and Fraenkel (2012) also demonstrated the existence of inattentional deafness by presenting participants a short video of two women wrapping up a present, two men preparing food and drink, and an additional man walking through the left side of scene, where two men at, while repeatedly saying “I am a gorilla.” Half participants were asked to listen to two men’s conversation and the other half listen to two women’s conversation before watching the video. Two questions were asked to all participants about whether they noticed the walking gorilla, if not, whether they heard an unusual sound. The result indicated that 70% of the half participants who attuned to women’s conversation failed to report the presence of that additional man repeating “I am a gorilla,” which also supported the existence of inattentional deafness.

Lavie’s load theory can explain the mechanism of inattentional deafness as well. Similar to how it explains the mechanism of inattentional blindness, inattentional deafness occurs when the primary task accompanies with high perceptual load and the state of focused attention is maintained. In other words, irrelevant verbal information cannot be perceived because of the exhausted perceptual load. Under the perspective of distraction, the proposed distraction capacity system provides an alternative explanation. The irrelevant verbal information is automatically resisted since its distractive influence does not exceed distraction capacity, which favors the maintenance of focused attention on the primary task.

### Distraction capacity is a dynamic system

In reviewing studies on inattentional blindness and inattentional deafness, a noteworthy phenomenon emerges: the distractive influence of the same distraction varies depending on the level of focused attention. Specifically, as greater attention is allocated to a primary task, susceptibility to distractions diminishes. This observation suggests that distraction capacity is not a static trait but a dynamic one. For instance, when deeply engaged in solving a math problem, particularly as one approaches its resolution, distractions such as the sound of a doorbell or feelings of hunger are less impactful. Conversely, during the initial stages of solving the problem, these distractions are more likely to disrupt focus. In this scenario, changes in the level of focused attention correspond to changes in susceptibility to distractions.

The concept of dynamic distraction capacity is supported by both attention control theory and the concept of motivational interference. According to Eysenck et al. (2007), attention control theory posits that anxiety significantly influences susceptibility to distraction. Individuals experiencing high anxiety often exhibit increased vulnerability to distractions and are more prone to multitasking behaviors. In contrast, lower levels of anxiety enable better focus on primary tasks. This variability implies the potential existence of a dynamic distraction capacity that fluctuates with emotional and cognitive states.

The concept of motivational interference, introduced by Fries and Dietz (2007), suggests that the presence of readily available and attractive leisure alternatives increases susceptibility to distraction and reduces motivation for the primary task. Thus, the level of motivational interference may also modulate susceptibility to distractions, reflecting that anxiety may not be the sole psychological factor affecting this susceptibility. So, the dynamic distraction capacity can be conceptualized as a mediator between anxiety or motivational interference and the susceptibility to distraction, proving it is dynamic in nature instead of fixed.

Furthermore, the dynamic distraction capacity is also supported by the most extreme example, the state of flow. The concept of flow was first proposed by Csikszentmihalyi (1990), representing the full immersion in a primary task, minimizing distractions, increasing enjoyment, and losing track of time. Although the state of flow is extremely hard to achieve, the professionals from different fields indicated frequent experience of the state of flow, such as surgeons operating surgeries, musicians playing instruments, and dancers dancing on the stage (Alameda et al., 2022; Nakamura and Csikszentmihalyi, 2002). Achieving the state of flow during learning has been considered as the most effective and efficient approach of learning, so helping students to achieve the state of flow and thereby improve their academic performance have been one of educators’ priorities (Harris et al., 2023; Jinmin and Qi, 2023). Admittedly, it is always difficult to achieve the state of flow with all the distractions from surrounding environment and the irrelevant thoughts from our minds (Schmidt et al., 2014). For example, students may hear the noise from the living room, receive notifications from social media applications, and glance at the basketball under the desk which distracts them from learning and triggers task-irrelevant thoughts. While exposing to such distractions, maintaining focused attention on learning is hard for students, not even to mention their minds are restlessly wandering (Smallwood and Schooler, 2006). When in the state of flow, individuals appear to completely resist distractions such that these disruptions no longer impair task performance. This observation raises an important question: What changes occur that transform the experience from one of the severe distractive influences on one in which distractions have no impact? I argue that distraction capacity is a dynamic system that is both expandable and shrinkable instead of a system with a fixed capacity. The dynamic distraction capacity perfectly explains the increased performance in resisting distractions when the state of flow is achieved. More specifically, achieving the state of flow accompanies with the maximal distraction capacity that automatically resists all perceived distractions, whereas achieving the state of focused attention accompanies with relatively smaller distraction capacity. Predictably, as less attention has been paid to the task, the distraction capacity shrinks correspondently, which leads to being distracted more easily.

Substantial studies from different fields support the dynamic feature of distraction capacity. In the field of mental fatigue, studies found people with mental fatigue are more likely to have difficulties with maintaining focused attention and experiencing distractive influence from distractions (Boksem et al., 2005; Faber et al., 2012; Shinar, 2017). The increased susceptibility of distraction during mental fatigue may lead to the decreased distraction capacity. For example, a student who studied for 3 h may not be able to resist the temptation of watching some videos for relaxation even though such distraction was able to be resisted at the beginning of studying, which reflects the changes in distraction capacity, from capable of resisting task-irrelevant thoughts to fail to resist. Furthermore, more evidence has been found in the studies related to boredom. Experiencing boredom also leads to the diversion of attention from primary task to a distraction (Gupta and Irwin, 2016; Mann and Robinson, 2009; Struk et al., 2016). For example, a student who has no interest in studying mathematics is less likely to resist the temptation of playing video games compared to studying an interested subject, which indicates the distraction capacity changes based on the experience of boredom. Additionally, the changes in distraction capacity may be attributed by other constructs, such as anxiety, stress, motivation, or urgency (Eysenck and Calvo, 1992; Fries and Dietz, 2007; Reinholdt-Dunne et al., 2009). Overall, the dynamic feature of distraction capacity resolves the conflicts in understanding the varied heaviness of distractive influence of the same distraction under different conditions, which provides an alternative explanation of the cognitive process of distraction and enriches our understanding of distraction.

### Exceeding the distraction capacity

Exceeding the distraction capacity results the conscious awareness of the distraction. More specifically, distraction occurs when the limited distraction capacity is no longer able to automatically resist all the perceived distractions, thereby diverting attention from primary task to distraction and requiring further cognitive processing. For example, a student closes the bedroom door and puts on a noise-canceling headphone for minimizing the distractive influence from the TV noise in the living room, thereby better maintaining focused attention on studying mathematics. In this example, the primary task is studying mathematics, and the distractive influence from TV noise exceeds the distraction capacity as the student becomes consciously aware of the distraction and takes active actions. Although the distraction capacity is dynamic based on the level of focused attention, it still has a momentary limitation in capacity that can be exceeded. Therefore, the exceedance of distraction capacity and conscious awareness of distraction suggest the fact of being distracted, leading to the attention control system.

### The evolutionary psychology perspective of distraction capacity

From an evolutionary psychology perspective, distraction capacity can be conceptualized as a developed cognitive subsystem that enhances the ability to achieve focused attention. This subsystem likely played a critical role in the survival of our ancestors, enabling them to prioritize essential stimuli in life-threatening situations. Gazzaley and Rosen (2016) illustrate this concept with a vivid example: our ancestors inhibit their perceptions to decrease goal interference for maximally detecting the hidden Jaguar’s low, grumbling snarl (p. 32). Such a subsystem minimizes interference from irrelevant stimuli, allowing maximum attention to life-preserving cues.

The development of distraction capacity represents a cost-efficient approach to achieving focused attention and task completion. First, focused attention can never be achieved if distractions continuously intrude. Both partial attention and divided attention are widely discussed concepts in the field of attention, representing part of our attention resource is occupied by task-irrelevant information, which hinders the maintenance of focused attention (Nadel, 2003; Rose, 2011). Therefore, resisting distractions becomes a necessary cognitive process for achieving focused attention. Second, achieving focused attention most effectively involves an automatic, low-effort system for managing distractions. Empirical studies emphasize that distraction inhibition often relies on active, conscious control, while the unconscious processes underlying focused attention remain underexplored (Draheim et al., 2022; Eysenck et al., 2007; Hopfinger et al., 2000). The development of such an automatic and efficient system can be understood as an evolutionary adaptation for prioritizing focused attention while maintaining the flexibility to respond to critical stimuli. This subsystem not only supports efficient task performance but also safeguards survival in dynamic and potentially dangerous environments.

### Theoretical distinction between distraction capacity and relevant attention constructs

Phenomena such as the irrelevant sound effect, inattentional blindness, and inattentional deafness exemplify the unconscious side of attention. The proposed construct of distraction capacity inevitably overlaps with related attention concepts—such as unconscious attention, involuntary attention, and automatic processing—because all address aspects of how information or stimuli can be processed without conscious awareness (Posner and Snyder, 2004; Schneider and Shiffrin, 1977). However, despite these conceptual similarities, *distraction capacity* is theoretically distinct from these existing attention constructs for three key reasons.

First, distraction capacity is developed from the perspective of distraction rather than from the perspective of attention. These two perspectives differ fundamentally in cognitive orientation. The attention perspective seeks to explain how the mind selects, prioritizes, and sustains processing of goal-relevant information or stimuli, whereas the distraction perspective examines how irrelevant or competing stimuli interfere with, interrupt, or divert attention away from the primary task (Johnson and Proctor, 2004; Wang, 2022). Emphasizing the distraction perspective in this manuscript highlights the cognitive processes by which distraction pulls attention away from a primary task, rather than framing the phenomenon as an attention shift toward distraction as viewed from the attention perspective.

Second, distraction capacity is defined on the premise that a primary task is actively in progress and requires the full allocation of attentional resources. This premise establishes a clear boundary between distraction capacity and other attention constructs. In unconscious attention or automatic processing, certain stimuli are processed without awareness regardless of whether a primary task is being performed. For instance, Abrams and Christ (2003) found that sudden motion in the periphery captures attention automatically, even when participants are not engaged in a primary task. Therefore, the primary task is not required for the cognitive mechanism of these relevant attention concepts. In contrast, the concept of distraction capacity has limited applicability in the absence of a primary task. Being distracted during tasks that do not require intensive attention—such as casually watching television or engaging in light exercise— conceptually differs from being distracted during tasks that demand full attentional resources (Eyal, 2019).

Third, the underlying mechanism differs. Distraction capacity refers to the unconscious resistance of task-irrelevant information and stimuli in order to maintain focused attention on the primary task. In contrast, related attention concepts describe the unconscious processing of task-irrelevant information and stimuli. The classic Cocktail Party Effect proposed by Cherry (1953) demonstrates that even when engaged in conversation, some attentional resources remain available to process certain task-irrelevant stimuli, such as hearing one’s name. However, when the premise of distraction capacity is met—that is, a primary task demands full attentional resources—the focus shifts from merely processing irrelevant stimuli to resisting them, thereby enhancing task performance. While theoretically plausible, the distraction capacity system requires empirical validation to confirm its functional mechanisms.

### The limitation of distraction capacity

Similar to the concept of attention capacity or working memory capacity, the distraction capacity is also a limited capacity for resisting perceived distractions, which means not all perceived distractions can be resisted while focusing on a task (Baddeley and Hitch, 1974; Kahneman, 1973). As discussed above, I conclude that we can automatically resist visual and verbal information that has limited distractive influence, such as background music, other’s conversation, or a walking gorilla, while we focus on a task. However, the distraction capacity is not the panacea for us to automatically resist every distraction we perceive, which is also the reason of why we can stay in the state of focused attention forever. The widely discussed changing-state effect is a representative example of how distraction capacity is exceeded and how attention is captured (Hughes et al., 2007; Meinhardt-Injac et al., 2022). Meanwhile, it is important to notice that distraction capacity is a cognitive system that only functions unconsciously. Distraction is not limited to the visual or verbal information from external environment, the unexpected interruptions, mind-wandering, or multitasking are all different types of distraction which cannot be unconsciously resisted by distraction capacity, which leads to the wonder of another cognitive system that inhibits such distractions and maintains focused attention (May and Elder, 2018; Pachai et al., 2016; Wang, 2022).

### Practical measurements of distraction capacity

Although the existence of a dynamic distraction capacity is theoretically plausible, empirical validation is essential to establish its functional significance. Since the contextual setting of the proposed model is educational setting, measuring distraction through task performance and attention stability during learning activities is the most ideal.

One practical method for measuring distraction is the systematic manipulation of background music during the execution of a primary cognitive task. Extensive studies have demonstrated the detrimental effects of background music on academic learning and information processing (Anderson and Fuller, 2010; Brodsky and Slor, 2013; Furnham and Strbac, 2002; Goltz and Sadakata, 2021). The cognitive mechanisms of background music on learning vary across different attention models, ranging from increased cognitive load to competition for attentional resources (Kahneman, 1973; Lavie et al., 2004). There is a broad consensus that background music consumes attentional resources for processing auditory information, thereby diminishing the resources available for processing the primary task. Within this framework, distraction capacity can be measured to the extent to which an individual can sustain stable task performance as the intensity of background music increases. For example, participants may be instructed to engage in a reading comprehension task—such as reading a chapter from a cognitive psychology textbook—while background music begins at a low volume and is gradually increased. Following the reading phase, participants would complete a free recall or comprehension test. Maintaining stable performance despite increasing auditory interference indicates a high level of distraction capacity, whereas performance decline at lower intensity levels signifies greater susceptibility to distraction and reflects a lower distraction capacity.

Another practical measurement of distraction capacity involves quantifying distraction capacity by assessing the number of concurrent task-irrelevant stimuli that can be resisted without observable performance decline. In daily life, task-irrelevant distractions are prevalent and consistently trigger task-irrelevant thoughts, thereby compromising focused attention on the primary task (Kane et al., 2021; Wong et al., 2022; Xie et al., 2016). For instance, students may overhear others’ conversations, attracted by some book titles, or alerted by the notification sound from smartphone while studying in a library. Since the types of distraction in educational settings are limited, a standardized criterion can be developed for systematically quantifying the maximum number of distractions that can be resisted without detriments the performance of primary task.

Establishing psychometrically validated criterion of distraction capacity not only allows for standardized assessment across individuals but also enables application in educational settings.

## The attention control system

The attention control system constitutes the second cognitive component of the proposed dual system mode, designed to represent our conscious responses to the distractions that exceed the distraction capacity. As previously discussed, distraction capacity refers to the unconscious resistance to distractions, whereas attention control represents the conscious inhibition of distractions once distractions are recognized. However, unlike the conventional understanding of attention control under the perspective of attention, the proposed attention control system introduces a novel understanding by examining attention control under the perspective of distraction. This perspective offers a unique angle for exploring the cognitive processes involved in distraction.

In essence, the attention control system is a top-down cognitive process that governs the decision to either refocus on the primary task or engage with the distraction after becoming consciously aware of it. Importantly, the sources of distraction are not limited to perceived verbal and visual stimuli but also include internally generated, task-irrelevant thoughts. To clarify how attention control system functions, this paper begins with a short summary of the current understanding of attention control. It then discusses the pivotal role of the proposed attention control system within the comprehensive cognitive process of distraction. Finally, the factors influencing the decision-making of the attention control system are explored based on their influence on attention control.

### Current understanding of attention control

Attention control is a foundational cognitive ability that has been extensively conceptualized through multiple frameworks. One prominent perspective situates attention control within Baddeley’s multicomponent model of working memory, where it is viewed as a critical function of the central executive. This role involves processing relevant information while inhibiting interference from irrelevant stimuli (Baddeley, 2002; Baddeley and Hitch, 1974; Logie, 2016). Meanwhile, attention control is also an important component of executive functions for supporting goal-directed behavior by managing distractions and maintaining focus (Clements et al., 2016; Diamond, 2013; Miyake et al., 2000). Beyond its cognitive dimensions, attention control plays an important role in self-regulation as well (Norman and Shallice, 1986; Zimmerman, 2000). For example, the classic marshmallow test by Mischel et al. (1972) demonstrated that delaying gratification requires sustained attention control to resist immediate impulses. This connection between attention control and self-regulation highlights its role in aligning behavior with long-term goals. Expanding on this, Shipstead et al. (2016) explored the underlying system of attention control and introducted two important mechanisms, intentional maintenance mechanism and intentional disengagement mechanism, illustrating attetion control inolves the active act of focusing on the primary task and removing irrelevant informtion. By synthesizing the theoretical understanding of attention control, Oberauer (2024) summarized that attention control is the ability to prioritize those representations that are relevant for the person’s current goal, thereby enabling them to think and act in accordance with their intentions. Therefore, the essence of attention control can be understood as the effortful regulation of cognitive resources to maintain focus on tasks and resist distractions.

Numerous cognitive tasks have been specifically designed to assess individuals’ ability to control their attention. Among these, the classic Stroop Task is one of the most widely used cognitive tasks for evaluating attention control. For instance, Kane and Engle (2003) investigate the relationship between attention control and working memory capacity, demonstrating that individuals with higher working memory capacity exhibited greater resistance to interference, highlighting the interplay between these two cognitive systems. In addition to the Stroop Task, other cognitive tasks have been employed to capture different facets of attention control and distraction resistance. The Flanker Task assesses the ability to suppress interference from surrounding stimuli while focusing on a central target, while the Go/No-Go Task measures response inhibition by requiring participants to perform an action in response to specific stimuli while refraining from acting on others. Findings from these cognitive tasks significantly contribute to the understanding of attention control and relative cognitive constructs.

Substantial evidence from neuroscience supports the cognitive mechanisms underlying attention control, highlighting its critical role in regulating behavior and achieving goal-directed tasks. At first, the prefrontal cortex (PFC) has been widely recognized as indispensable for attention control, executive functions, working memory, and self-regulation because it involves in cognitive control processes, enabling individuals to prioritize goal-relevant information, inhibit distractions, and adapt to changing task demands (Baddeley, 2003; Banfield et al., 2004; Menon and D’Esposito, 2022; Miller and Cohen, 2001; Rossi et al., 2009). To further understand the mechanisms of attention control, Petersen and Posner (2012) roposed three key components of the attention system: the alerting network, responsible for maintaining a state of vigilance; the orienting network, which facilitates the selective allocation of attention to relevant stimuli; and the executive network, involved in resolving conflicts and maintaining task goals. Focusing on the orienting network, two important networks, dorsal attention network (DAN) and ventral attention network (VAN), have been found (Corbetta and Shulman, 2002). The DAN, primarily associated with top-down attention control, allows individuals to voluntarily direct focus based on task goals, engaging brain regions such as the intraparietal sulcus and frontal eye fields. In contrast, the VAN supports bottom-up attention control, automatically redirecting focus to salient or unexpected stimuli, and involves areas like the temporoparietal junction and ventral frontal cortex (Corbetta and Shulman, 2002; Vossel et al., 2014). In addition, the default mode network (DMN) provides further insight into the neural dynamics of attention control. The DMN, encompassing the ventral and medial regions of the frontal and parietal cortices, typically exhibits deactivation during active task engagement when attention is intentionally directed toward external goals. Conversely, the DMN becomes active during internally directed, leisure-oriented, or introspective activities, suggesting its role in facilitating shifts between task-focused and self-referential thought processes (Andrews-Hanna et al., 2014; Buckner et al., 2008). Together, these findings underscore attention control as a cognitive ability that involves voluntary (top-down) processes.

### The significance of attention control system

The attention control system proposed within the dual system model of distraction presents a nuanced departure from the conventional understanding of attention control. Unlike the current understanding of attention control, which emphasize the sustained allocation of attentional resources to a primary task, the proposed attetnion control system highlights the cogntivie process of refocusing on primary task or being distracted after consciously aware the distraction. More specifically, the attetnion control system centers on the decision-making processes involved in either refocusing on the primary task or shifting to the distraction. For example, consider a student is focusing on watching the recorded lecture video in the dormitory. While the student may successfully ignore background noise, such as the sounds of peers walking or talking outside, the conscious awareness of a loud knock at the door becomes unavoidable. This leads to a decision-making: refocusing on watching the lecture video and ignore the fact that someone is knocking the door, or answering the door. In this scenario, watching the lecture represents the primary task, while the door-knocking constitutes the distraction. The distraction capacity is exceeded after consciously awre of the knocking sound, and entering the attetnion control system when comes to the decision-making.

From the perspective of attention, attention control is viewed as a continuous process demanding mental effort to maintain focused attention on a primary task (Kahneman, 1973). With the needed attentional resources allocated to the primary task, the focused attetnion is maintained and distractions are resisted. Conversely, the attention control system, framed through the perspective of distraction, focuses on how attention is redirected following the inevitable conscious awareness of a distraction. This distinction underscores a fundamental difference in how attention control is conceptualized.

### Factors influence attention control and its relevance to attention control system

Various factors have been widely acknowledged to significantly influence attention control performance. One prominent factor is working memory capacity, which is strongly linked to attention control. Research consistently demonstrates that individuals with higher working memory capacity exhibit superior performance in maintaining goal-relevant information while effectively ignoring irrelevant distractions (Kane and Engle, 2003; Oberauer, 2019; Unsworth and Spillers, 2010). Similarly, executive function is another critical cognitive construct associated with controlling attention and inhibiting distractions. For instance, Miyake et al. (2000) identified executive function as essential for successful attention control. Valcan et al. (2020) conducted a longitudinal study that revealed executive function as a strong predictor of children’s future performance in mathematics, reading, and writing, underscoring its role in enhancing learning outcomes through improved attention control and distraction inhibition. In addition to these cognitive constructs, emotional states also play a pivotal role in attention control. The attention control theory proposed by Eysenck et al. (2007) suggests that anxiety impairs attention control by weakening top-down processes while increasing vulnerability to bottom-up influences, leading to difficulties in maintaining focus on goal-relevant tasks and resisting irrelevant distractions. Meanwhile, negative mental states, such as fatigue, have been shown to lower the performance of attention control and distraction resistance. Calderwood et al. (2014) found that fatigue and other negative moods are positively correlated with multitasking behaviors during homework completion, reflecting challenges in maintaining focused attention on primary tasks.

Although the attention control system is conceptually distinct from attention control, they share the same underlying cognitive process, the top-down cognitive process (Corbetta and Shulman, 2002; Draheim et al., 2022). Consequently, the factors that influence attention control performance may also affect decision-making within the attention control system. More specifically, these factors may have various influences on the decision of whether refocusing on the primary task or shifting attention to distractions. For example, A highly anxious student may feel reluctant to proceed with completing an assignment and instead favor shifting attention to distractions, such as scrolling through TikTok. This preference for distraction reflects a top-down cognitive decision influenced by emotional and cognitive states. Conversely, if the assignment deadline is imminent, the student is more likely to consciously choose to refocus on the task despite the temptation of distractions. This example illustrates how decision-making within the attention control system can be influenced by anxiety and other factors, ultimately determining whether attention is maintained on the primary task or diverted.

### Summary

Conceptually, the attention control system is different from widely recognized understanding of attention control. It stresses the cogntivie process of refocusing on primary task or being distracted after being consciously aware of the distraction under the perspective of distraction, which is more appropriate to be framed as a decision-making process rather than a effortful control process under the perspective of attention. Thus, it is reasonable to conclude that being distracted is the result of a voluntary decision made by individuals. Similarly, choosing to ignore a consciously perceived distraction and refocus on the primary task is also a voluntary decision made by individuals. However, various factors—such as anxiety, fatigue, working memory capacity, and executive function—may influence this decision-making process, just as they affect attention control.

## Future directions and applications

Several future research directions and practical applications related to the proposed dual system model of distraction remain to be explored. The most pressing priority is the empirical validation of distraction capacity, as it constitutes the foundational construct of the distraction capacity system and, by extension, the conceptual cornerstone of the entire model. While the present work has provided extensive indirect evidence and theoretical inferences supporting the plausibility of its existence, the field still lacks direct, systematic, and replicable empirical demonstrations. Establishing such evidence would not only strengthen the model’s theoretical credibility but also provide a measurable construct for subsequent experimental and applied research. Equally important is the systematic examination of the attention control system, the second core system of the dual system model. The construct of attention control has been extensively studied in relation to working memory capacity (Engle, 2002; Kane and Engle, 2003) and executive control (Unsworth and Spillers, 2010), and its validation within the proposed framework is essential for achieving both internal coherence and external recognition of the model. Future investigations should examine both systems independently and in their dynamic interaction, allowing researchers to determine how distraction capacity and attention control jointly influence performance across diverse contextual settings.

Another important future direction for the proposed model is the practical measurement of distraction capacity. Although distraction capacity is inherently dynamic and subject to individual differences, quantifying it at the individual level can provide valuable insights into a person’s susceptibility to distraction. For instance, certain students may require a quieter and more controlled learning environment due to heightened vulnerability to distractions, whereas others may sustain focused attention even in an environment with substantial background noise. Awareness of one’s distraction capacity can guide the development of personalized strategies for structuring learning environments, enabling individuals to manage cognitive demands effectively and minimize the risk of exceeding their distraction capacity.

Given that distraction capacity is inherently dynamic, advancing our understanding of the factors that influence distraction capacity is of critical importance. While prior research has identified several variables, including motivation, fatigue, task engagement, and environmental complexity, as significant factors that impact on the *distraction capacity system* proposed in the current model remains to be empirically established (Boksem et al., 2006; Boksem and Tops, 2008; Griffin, 2014). Rigorous empirical investigations are therefore needed to determine which factors most effectively enhance individuals’ distraction capacity, thereby enabling them to sustain attention on a primary task despite the presence of competing stimuli. To strengthen individuals’ focused attention on a primary task, future research should systematically examine which factors most effectively increase individuals’ distraction capacity and explore the underlying mechanism. Such inquiry holds particular relevance in educational settings, where optimizing distraction capacity could substantially improve students’ capacity to maintain focused attention during learning activities.

Equally important is the systematic examination of factors influencing the attention control system. Specifically, future research should explore the factors that facilitate rapid reorientation toward the primary task following the perception of distractions, as well as the factors that increase susceptibility to prolonged diversion of attention. Identifying these factors that either promote attentional control or foster indulgence in distractions would yield critical insights into attention control system. This knowledge would be especially valuable in educational contexts, where strengthening students’ ability to control attention, inhibit task-irrelevant processing, and re-engage with learning tasks could lead to significant improvements in learning efficiency and academic performance (Draheim et al., 2022; Mitchell et al., 2008; Oberauer, 2024).

## Conclusion

The establishment of the dual system model of distraction provides a significant advancement in conceptualizing distraction as a distinct yet complementary construct to attention. By systematically reviewing both attention models and distraction-related models, the theoretical linkage between distraction and attention has been critically re-examined, revealing that these constructs are not opposite but operate within the same limited capacity framework of attentional resources. Distraction arises when insufficient attentional resources are allocated to the primary task, resulting in a shift of attention toward competing stimuli.

The proposed model extends existing attention theories by addressing the cognitive mechanisms of distraction from the perspective of distraction, thereby filling a long-standing theoretical gap. It consists of two cognitive systems: the distraction capacity system, representing the unconscious and automatic resistance to task-irrelevant stimuli, and the attention control system, representing the conscious, conscious decision-making process that occurs once a distraction exceeds distraction capacity. By articulating both the unconscious and conscious processes of attention, the model offers a more comprehensive account of distraction than frameworks that focus exclusively on attention. It addresses critical phenomena such as inattentional blindness, inattentional deafness, the state of flow, and variability in distraction susceptibility, providing a theoretically grounded explanation of the cognitive mechanism of distraction.

In conclusion, the dual-system model of distraction establishes distraction as a meaningful cognitive construct with distinct mechanisms. It provides a coherent theoretical basis for understanding how individuals navigate between focus and distraction, offering a structured account that can guide future conceptual and empirical work in this underdeveloped yet essential domain.

## Data Availability

The original contributions presented in the study are included in the article/supplementary material, further inquiries can be directed to the corresponding author.
